# Transient increase in flicker ERG amplitude preceding OCT-detected macular thickening in eyes with acute anterior uveitis

**DOI:** 10.1007/s10384-026-01383-8

**Published:** 2026-05-30

**Authors:** Kumiko Kato, Keitaro Mizumoto, Ryunosuke Nagashima, Mayumi Momose, Yuzen Kashima, Sumine Mori, Hisashi Matsubara, Mineo Kondo

**Affiliations:** Department of Ophthalmology, Mie University Graduate School of Medicine, 2-174 Edobashi, Tsu, Mie 514-8507 Japan

**Keywords:** Flicker ERG, Acute anterior uveitis, Amplitude, Inflammation, Central macular thickness, OCT

## Abstract

**Purpose:**

To determine the changes in amplitude of the flicker electroretinograms (ERGs) and central macular thickness (CMT) during the acute phase of acute anterior uveitis (AAU).

**Study design:**

Retrospective case series.

**Methods:**

Flicker ERGs (RETeval) and optical coherence tomographic (OCT) images were recorded from the affected and fellow eyes at multiple examination times. The outcomes are expressed as a ratio of the affected-to-fellow eyes (×100). The time courses were modeled by polynomial regression (degrees 1–4) with model selection based on R^2^, Akaike, or Bayesian information criteria (AIC, BIC), and the *P* values. The peak timing and uncertainty were estimated by patient-level cluster bootstrap resampling (B=2000).

**Results:**

Eight eyes from eight patients (4 men, 4 women; mean age 48.8±15.8 years) underwent 3.3±1.0 paired ERG/OCT assessments. The ratio of the amplitudes of the two eyes had a significant nonlinear time course, and a 4th-degree model was best supported (R^2^=0.411, *P*=0.021). Bootstrap estimated an amplitude peak at 11.8 days (median 11.9; 95% CI 10.2–19.0) with a peak value of 134.6%. The ratios of the implicit times were not significantly associated with the disease duration. The CMT ratio had a significant nonlinear time course and a 2nd-degree model was best supported (R^2^=0.358, *P*=0.006). Bootstrap analyses estimated a CMT peak at 35.4 days (median 35.1; 95% CI 29.2–48.4) with a peak value of 108.6%.

**Conclusions:**

The flicker ERG amplitudes increase transiently around two weeks after the onset of AAU and subsequent macular thickening in the OCT images. These findings suggest that flicker ERGs may serve as an early functional retinal marker of subclinical posterior segment inflammation in eyes with AAU.

## Introduction

Acute anterior uveitis (AAU) is an inflammatory disorder that typically affects the anterior segment of the eye and is often unilateral [1]. In a retrospective cohort study conducted in Japan, AAU was found to be the most common form of anterior uveitis accounting for approximately 20% of all anterior uveitis cases [2]. Although the inflammation in AAU is generally confined to the anterior segment [3], severe or recurrent AAU cases can be complicated by cystoid macular edema (CME) [4]. Recent OCT studies report an increase in the retinal and choroidal thickness in the affected eyes compared to the fellow eye in cases of AAU [5, 6]. Analyses of swept-source OCTA images further demonstrate alterations in the vascular structures of the posterior segment in eyes with anterior uveitis [7]. However, the time course of the macular thickening after the onset of the signs and symptoms of AAU has not been determined.

Electroretinographic (ERG) abnormalities are reported in different types of retinal disorders including diabetic retinopathy [8] and retinal vein occlusion [9]. An increase in the implicit times is often detected in eyes with uveitis [10], and we have observed a transient increase in flicker ERG amplitudes in eyes with anterior segment inflammation after lens reconstruction surgery along with a time lag between the peak amplitude increase and the peak of increase in the central macular thickness (CMT) [11]. Given the similarities in the primary sites of inflammation, we hypothesized that AAU may also have a transient increase in the flicker ERG amplitudes and a temporal dissociation between the functional (ERG) and structural (OCT) changes. To the best of our knowledge, no previous study has characterized the temporal dissociation between the changes in the flicker ERGs and OCT-detected macular thickening in eyes diagnosed with AAU.

The purpose of this study was to determine the longitudinal changes in the amplitudes and implicit times of the flicker ERGs and OCT parameters in eyes with AAU. In addition, we developed a statistical model of the temporal patterns of the ERG and CMT changes during the resolution of the AAU

## Methods

### Study design and participants

This retrospective study was conducted on AAU patients examined in the Mie University Hospital between May 2018 and December 2020. Eyes were diagnosed with AAU if they had the following characteristics: 1) unilateral inflammation; 2) sudden onset; 3) non-granulomatous anterior uveitis; 4) no clinical or systemic findings suggestive of other uveitis entities based on systemic evaluation; and 5) no clinical evidence of retinal vasculitis and retinitis. After diagnosing AAU, the eyes were treated topically with betamethasone 6 to 8 times/day with the initial frequency of topical betamethasone determined by the severity of the anterior chamber inflammation on slit-lamp examination [12]. As the objective findings improved, the dosage of betamethasone was gradually reduced and then discontinued within 3 months after the symptom onset.

Fluorescein angiography was not routinely performed in this cohort and was reserved for cases in which posterior segment involvement or clinically apparent CME was suspected; no such cases were observed.

Ethical approval for this study was obtained from the Ethics Committee of Mie University Hospital (Approval No. 2021-078). The procedures used in this study conformed to the tenets of the Declaration of Helsinki.

This study included patients diagnosed with AAU who were followed until the resolution of AAU. The exclusion criteria included patients with bilateral AAU, diabetic retinopathy, retinal vein occlusion, and those who had a recurrence of the AAU during the study period.

### Data collection

Flicker ERGs were recorded using the RETeval system (LKC Technologies) under natural pupillary conditions. The intensity of the stimulus flash was 32 photopic Td-s, and ERGs were recorded with specialized skin electrodes placed approximately 2 mm below the lower eyelid margin. The following parameters were assessed: the implicit times and amplitudes of the flicker ERGs and the CMT. The CMT was measured on the images obtained by a spectral-domain OCT device (Spectralis; Heidelberg Engineering Inc.). The CMT was defined as the average retinal thickness within the central 1-mm area.

The amplitudes and implicit times of the flicker ERGs, and the CMT were expressed as ratios relative to the fellow eye: ratio (%) = (affected eye/fellow eye) × 100. This method was used because the pre-onset baseline measurements were unavailable in cases of acute-onset AAU and because the ERG measured values had substantial inter-individual variations. Interocular asymmetry in CMT is small in healthy adults (grand mean 5.6 μm, 95% CI 4.6–6.5, measured by Spectralis SD-OCT posterior pole asymmetry analysis) [13], supporting the use of the fellow eye as a practical reference for CMT. Likewise, in healthy subjects recorded under natural pupil conditions with the RETeval system at 32 photopic Td-s, the normal range (95% reference interval) of between-eye differences in flicker ERG amplitude is reported to be <12.8% [14], supporting fellow-eye normalization for ERG amplitude.

Because the examinations were not performed at specific time points, e.g., at 1 week or 1 month after the onset of AAU, the evaluation time was defined as the number of days from the onset of the symptoms (disease duration), and the outcomes were analyzed as a function of the disease duration.

### Statistical analyses

Because the onset of the AAU was unscheduled and the follow-up visits occurred at different times after the onset of the symptoms; the outcomes were modeled as continuous functions of days from symptom onset (disease duration) using polynomial regression. For each visit, the amplitudes and implicit times of the flicker ERGs, and the central macular thickness (CMT) in the affected eye were expressed relative to that of the fellow eye (affected/fellow ×100) to reduce the inter-individual variability and to focus on AAU-related inter-eye asymmetry.

Polynomial models of degrees 1 to 4 were fitted by the least squares method. Model adequacy was compared using the coefficient of determination (R^2^) and information criteria (Akaike information criterion [AIC], and Bayesian information criterion [BIC]). The degree with the lowest information criterion was considered the best-supported model, and adjacent degrees were examined as sensitivity analyses when AIC and BIC selected different models. The fitted curves were used to describe the temporal trends and to estimate the timing of the peak of the changes. To account for within-subject correlations arising from repeated measurements, cluster-robust standard errors at the patient level were applied. A two-sided *P* value <0.05 was considered statistically significant.

To estimate the implicit times and amplitudes of peak changes and their uncertainties, we performed a patient-level cluster bootstrap (B = 2000). In each bootstrap replicate, patients were resampled with replacements, and all observations from each selected patient were retained; the selected polynomial model was refitted to the resampled dataset. The peak day was defined as the day within the observed follow-up window (evaluated on a dense grid) at which the fitted curve attained its maximum, and the 95% bootstrap confidence interval (CI) was computed using the 2.5th and 97.5th percentiles of the bootstrap distribution. Peak-timing differences between amplitude and CMT were summarized from the bootstrap distribution of (amplitude peak day − CMT peak day). Because implicit times did not have a clear temporal trend in polynomial fits, the peak-timing inference was not emphasized for this outcome.

All analyses were performed using Python (version 3.13) with NumPy for polynomial fitting and Statsmodels for regression inference.

## Results

### Clinical characteristics of patients

Eight eyes of eight patients (4 men, 4 women) were studied. The mean age of the patients was 48.8 ± 15.8 years (range: 23-72 years). The mean number of ERG and OCT recordings/patient was 3.3 ± 1.0 (range: 2-5). The mean interval between the onset of the symptoms to the first ERG recording was 13.1 ± 10.5 days. Posterior synechiae were present in 5 of 8 cases. All were partial, and none had a pupillary deformation. The mean pupillary diameter at the initial visit was 3.0 ± 1.4 mm (range, 1.9–6.0 mm), and ERGs were successfully recorded from all cases.

### Representative case

**Figure 1:**
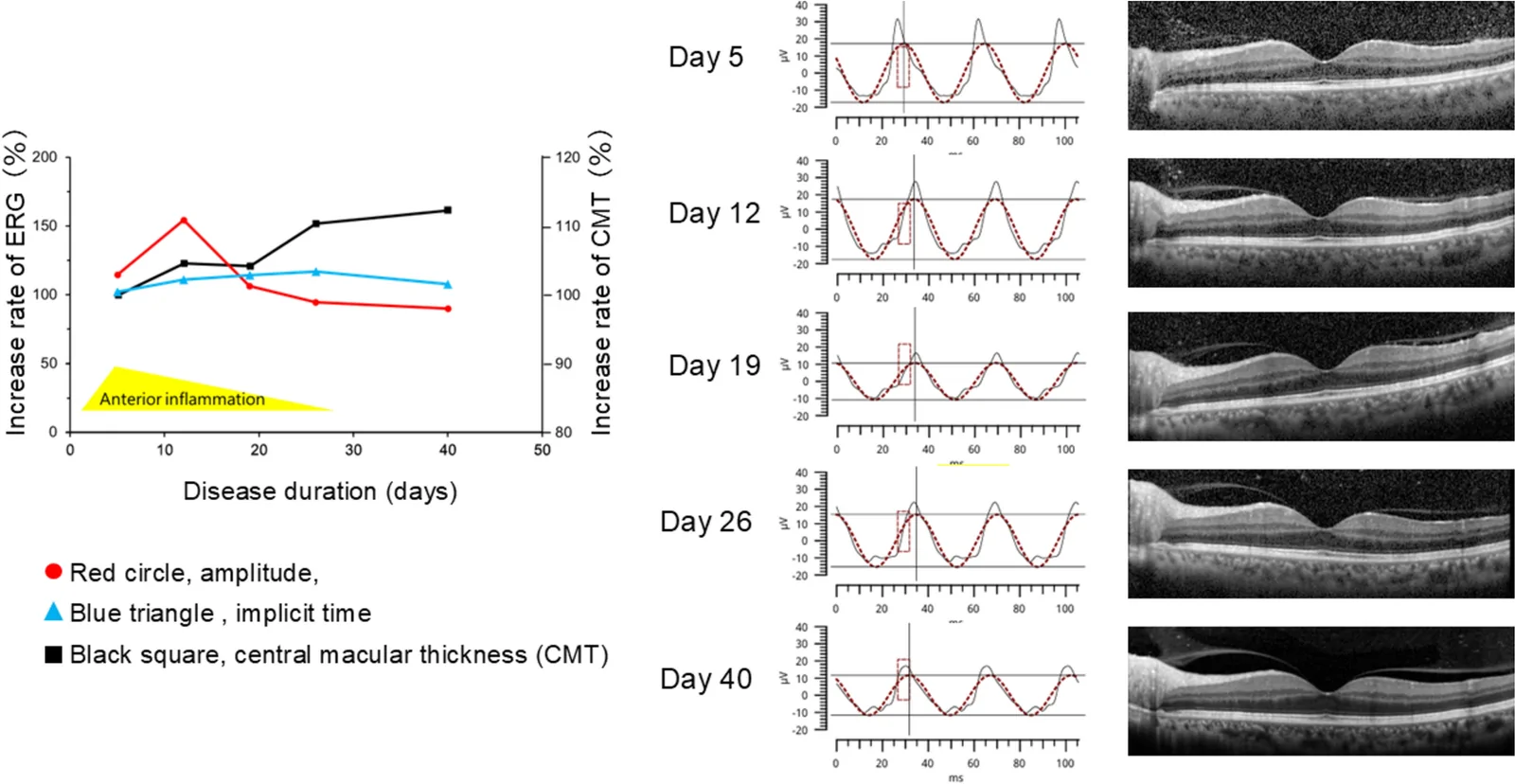
Changes of flicker electroretinogram (ERGs) and central macular thickness (CMT) in representative patients diagnosed with acute anterior uveitis (AAU). Left panel: The rate of the changes in the amplitudes and implicit times is represented on the left scale, and the rate of change of the CMT is shown on the right scale. The red circles and red line represent the amplitudes, the blue triangles and blue line represents the implicit times, and the black squares and black line represents the CMT. The amplitudes of the flicker ERGs peaked approximately two weeks after the onset of the signs and symptoms of AAU and decreased with treatment. The CMT increased after about one month from the onset. Middle panel: Changes in the flicker ERGs over the course of the AAU. The fundamental component (red dotted line) is superimposed on the reconstructed flicker ERG (solid black line) using the first 8 harmonics. Right panel: Changes in the CMT over the course of AAU. Although no obvious cystoid macular edema was observed, the retinal thickness at the fovea gradually increased.

The findings in a representative patient with AAU are shown in Figure 1. The patient presented 5 days after the onset of conjunctival injection, ocular pain, and marked anterior chamber inflammation in the affected eye. These signs and symptoms led to the diagnosis of AAU. OCT and flicker ERGs were recorded from both eyes, and parameters were analyzed as ratios relative to the fellow eye. At presentation, the CMT and flicker ERG parameters were comparable between the two eyes. Topical betamethasone was initiated six times/day. At the 1-week follow-up examination, the anterior segment inflammation had improved, while the flicker ERG amplitude ratio increased to 154% and the CMT ratio increased to 106%. At 2 weeks, the amplitude ratio returned to near baseline and anterior segment inflammation had largely resolved. At 3 weeks, the ratios of the amplitudes and implicit times were not significantly different, but the CMT ratio had increased to 110%. Systemic investigations were performed, and uveitis associated with systemic disease was ruled out.

Changes in the amplitudes and implicit times of flicker ERGs and central macular thickness.

**Figure 2:**
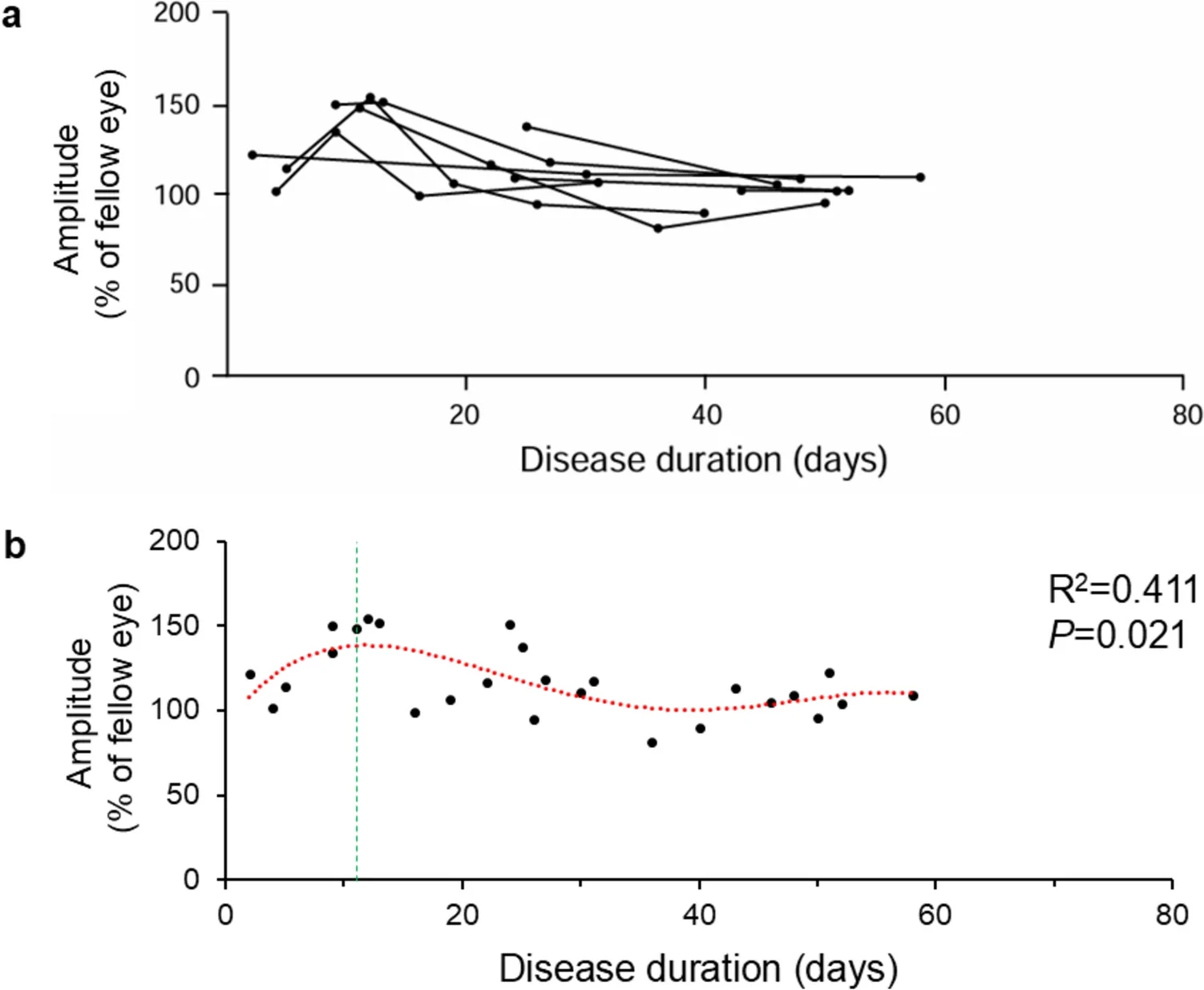
Changes in the flicker ERG amplitudes in eight patients with AAU. The rate of the amplitude increase relative to the fellow eye (affected/fellow ×100) is plotted against disease duration (days from signs and symptoms onset). a: Individual longitudinal profiles for each patient, illustrating the inter-individual variability in the amplitude changes over time. b: Pooled measurements with a fourth-degree polynomial fit. The fitted curve had an estimated peak at approximately 11.81 days after the onset of the symptoms (vertical green dashed line) (R^2^ = 0.411, *P* = 0.021).

**Figure 3:**
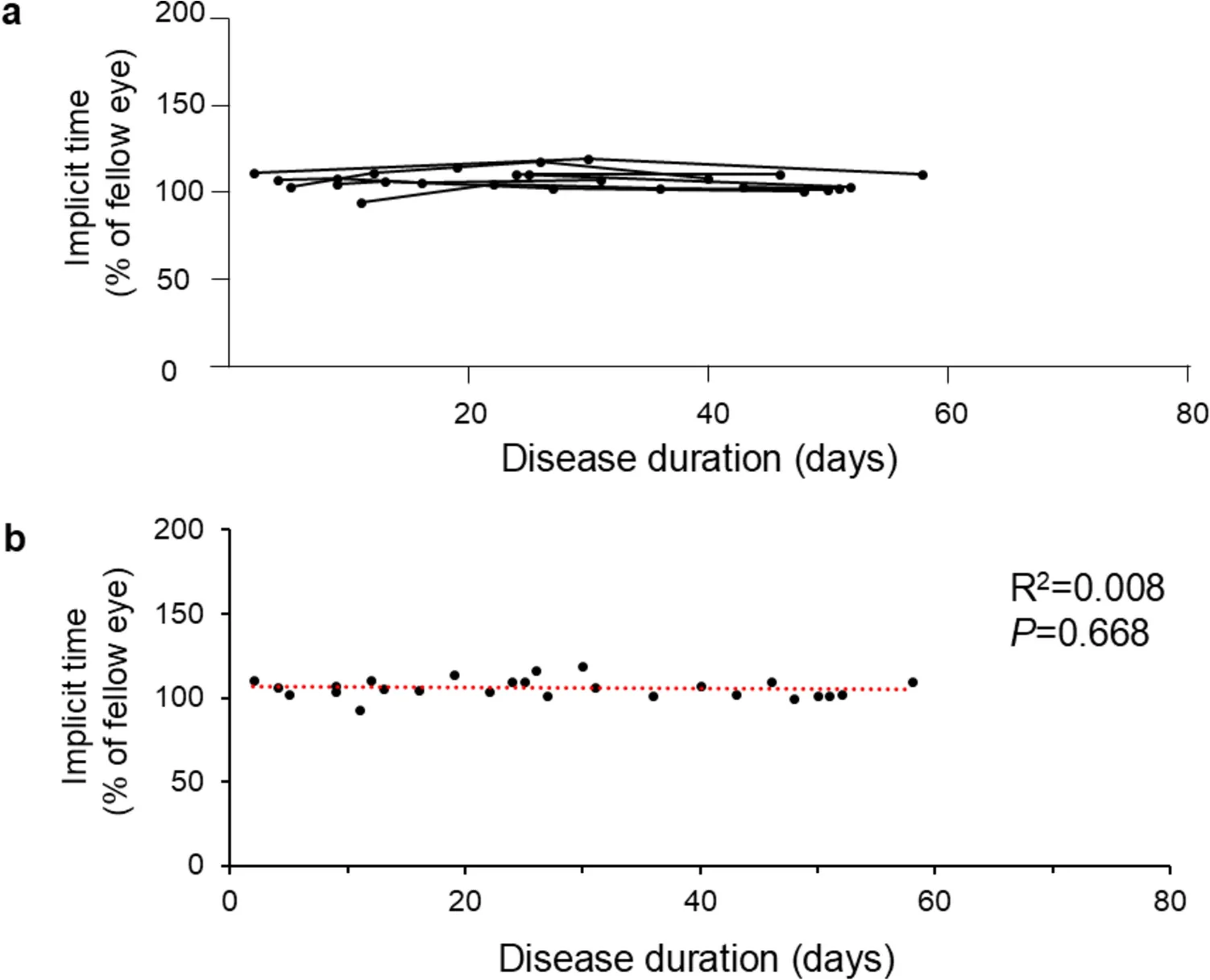
Changes in the flicker ERG Implicit times in eight patients with AAU. The implicit time ratio relative to the fellow eye (affected/fellow ×100) is plotted against the duration times (days from symptom onset). a: Individual longitudinal profiles for each patient. b: Pooled measurements with a linear fit (first-degree polynomial), selected as the best-supported model by Akaike, or Bayesian information criteria (AIC and BIC). No significant association with disease duration was observed (R^2^ = 0.008, P = 0.668).

**Figure 4:**
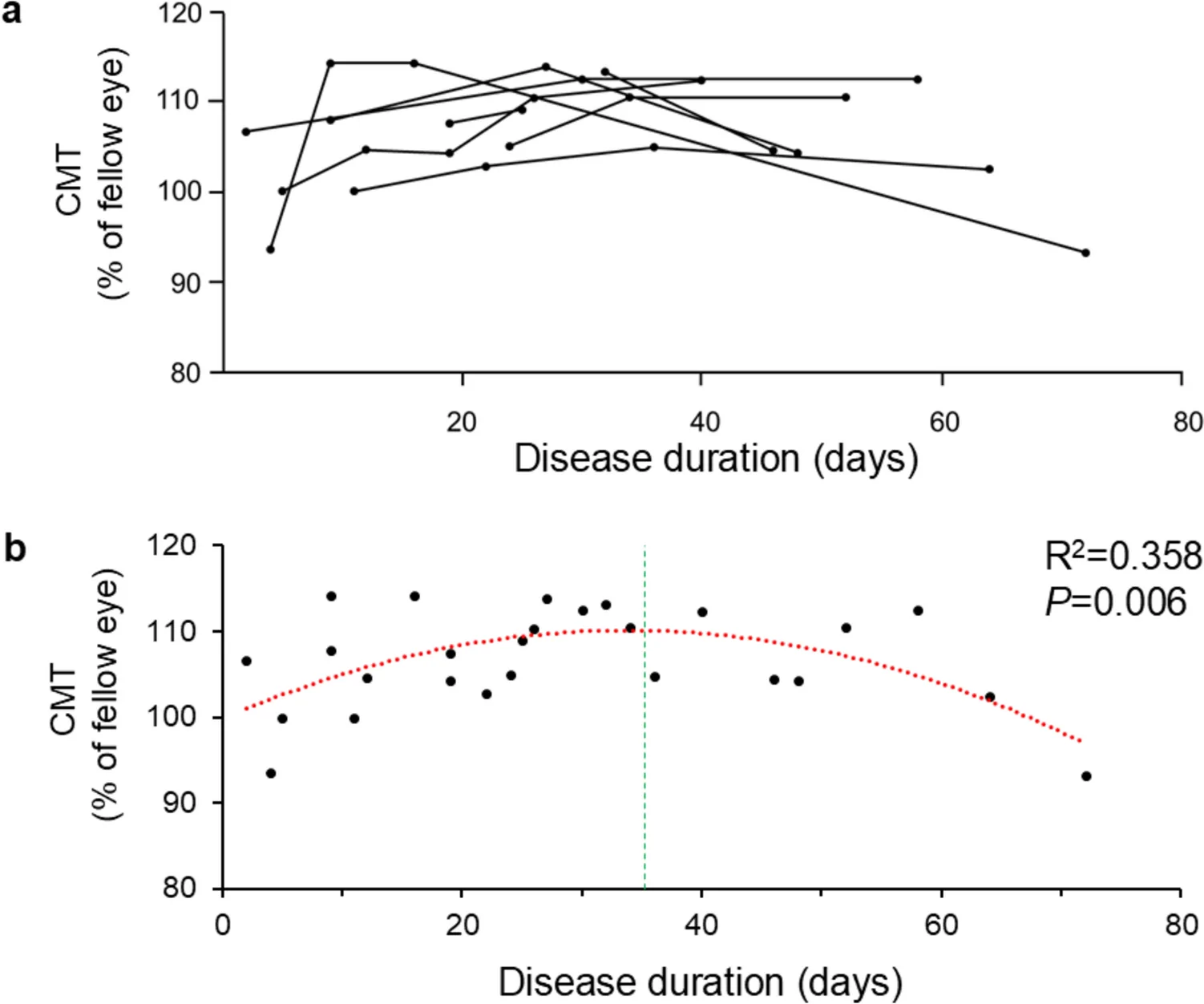
Changes in CMT in 8 patients with AAU. The CMT increase rate relative to the fellow eye plotted against disease duration. a: The individual data from each patient, shown by different lines and illustrates the variation in the CMT increase over time for each subject. b: Relationship between disease duration and CMT increase in AAU patients. The 2nd-degree polynomial regression model was selected based on the AIC and BIC values. A peak at 35.35 days (vertical green dashed line) after symptom onset (R^2^ = 0.358, *P* = 0.006) can be seen.

To visualize the individual variations, we plotted the changes in the test values for each case (Fig. 2A, 3A, 4A). No consistent change in the implicit times was detected during the observation period (Fig. 3A). However, many cases had an increase in the amplitude in the early stages of AAU (Fig. 2A), and the CMT tended to increase approximately one month after the onset of the AAU (Fig. 4A). When the test values of all patients were plotted, a non-linear relationship was suggested between the disease duration and either the amplitude or the CMT.

We analyzed the data for all patients using a polynomial regression model. For the amplitude ratio, the fourth-degree polynomial model provided the best-supported fit (highest R^2^ and lowest AIC, with BIC comparable to the third-degree model; Table 1). The overall model was significant (R^2^ = 0.411, *P* = 0.021; Fig. 2B). The fitted curve had an early peak in the amplitude ratios. In the patient-level cluster bootstrap analysis (B = 2000), the peak day was 11.8 days by point estimation with a bootstrap median of 11.9 days (95% CI, 10.20–18.96). The peak fitted value was 138.64% by point estimate (bootstrap median 140.52%, 95% CI 126.08–155.86).

**Table 1:** Polynomial Fit Results for Amplitude, Implicit Time, and Central Macular Thickness (CMT)

Degree of polynomial model	Amplitude				Implicit time				CMT			
	R^2^	AIC	BIC	*p*-value	R^2^	AIC	BIC	p-value	R^2^	AIC	BIC	*p*-value
1st-degree	0.214	228.1	230.6	0.017*	0.008	166.5	169	0.668	0.001	168.7	171.2	0.898
2nd-degree	0.215	230.1	233.9	0.062	0.056	167.2	171	0.517	0.358	159.2	162.9	0.006**
3rd-degree	0.345	227.4	232.4	0.023*	0.068	168.9	173.9	0.662	0.37	160.7	165.7	0.016*
4th-degree	0.411	226.6	232.9	0.021*	0.278	164.2	170.5	0.129	0.393	161.7	168	0.027*

*< 005, **< 0.01

AIC; Akaike information criterion, BIC; Bayesian information criterion.

AIC and BIC are commonly used to compare the goodness of fit between models, where lower values indicate better fit while accounting for the number of parameters used in the model. These criteria are particularly useful when comparing models with different complexities.

For the implicit times, no polynomial model had a significant association with the disease duration (Fig. 3B; Table 1), indicating no detectable time-dependent changes in these datasets.

For the CMT ratio, the second-degree polynomial model was best supported (lowest AIC and BIC; Table 1), and it was significant (R^2^ = 0.358, *P* = 0.006; Fig. 4B). In the patient-level cluster bootstrap analysis (B = 2000), the peak day was 35.35 days by point estimate, with a bootstrap median of 35.05 days (95% CI, 29.17–48.44). The peak fitted value was 108.63% by point estimate (bootstrap median 108.82%, 95% CI 106.77–111.31). In the bootstrap analysis, boundary hits were infrequent; peak-day estimates occurred at the lower/upper search boundaries in 0.0%/1.0% of replicates for the amplitudes (4th-degree; range 2–58 days) and 0.0%/0.7% for the CMT (2nd-degree; range 2–83 days). These findings suggested a minimal endpoint influence on the estimated peak timing.

Finally, bootstrap analysis of the peak-timing difference (amplitude peak day − CMT peak day) suggested that the amplitude ratio peak tended to precede the CMT ratio peak (median difference −23.17 days, 95% CI −36.76 to -11.82).

## Discussion

In these AAU cases, the flicker ERG amplitude ratio between the affected and non-affected eyes increased transiently and peaked at approximately 12 days after the onset of the signs and symptoms. However, the CMT assessed by the OCT images peaked at around 1 month. Thus, a temporal dissociation was observed between the functional changes and a structural change. Notably, the CMT peak remained <10% higher than of the fellow eye, suggesting that measurable macular thickening can develop even in AAU cases without clinically apparent CME. This time lag is particularly interesting because we had previously observed a similar dissociation between the amplitudes of the flicker ERGs increase and subsequent CMT increases after lens reconstruction surgery [11]. Given that both the AAU and postoperative inflammation after lens reconstruction primarily originate in the anterior segment, this shared temporal dissociation is especially noteworthy.

In uveitis, delayed implicit times are often reported as a typical ERG abnormality. However, in the present AAU cohort, the implicit time ratios did not have a significant time-dependent change, whereas the amplitude ratios did. This pattern is consistent with previous observations that transient amplitude enhancement may occur in conditions associated with relatively mild ischemia or mild intraocular inflammation, including preproliferative diabetic retinopathy [15] and non-ischemic central retinal vein occlusion [9]. Inflammatory diseases such as lens-induced uveitis [14], post-cataract surgery inflammation [11], and post-vitrectomy inflammation for epiretinal membrane [16] have demonstrated transient increase of the ERG amplitudes. In contrast, intense inflammation, such as in eyes with Vogt–Koyanagi–Harada disease, has been associated with delayed implicit times and reduced amplitudes in the acute phase [17].

Our cases had a significant increase in the amplitudes, but no significant delay in the implicit times. These findings suggest that the inflammatory effect in the present AAU cohort was relatively mild, which is supported by the absence of clinically apparent CME during the follow-up examinations.

The mechanism causing the inflammation-associated ERG amplitude enhancement remains incompletely understood. Experimental data suggest that inflammatory mediators, including nitric oxide, can modulate the ERG amplitude in a dose-dependent manner, and lower levels may enhance the ERG amplitudes [18]. However, higher levels may depress the responses [18]. Such pathways may plausibly contribute to the transient amplitude increase observed in AAU, although direct mechanistic validation in human AAU cases is beyond the scope of this study.

The delayed CMT peak may be related to the time required for secondary processes such as blood–retinal barrier dysfunction and fluid accumulation to develop. A similar delay is well recognized after cataract surgery in which the CME typically develops several weeks after the initial anterior segment inflammation [19]. Because the macular thickening likely requires a sufficient imbalance between vascular leakage and retinal fluid clearance to exceed a structural detection threshold, OCT-detectable changes may lag behind earlier functional alterations detected by ERG changes.

From a clinical perspective, the present findings suggest that flicker ERGs may complement slit-lamp and OCT assessments by examining an early functional marker that could inform monitoring during treatment and tapering, particularly when posterior segment involvement is not obvious during a routine examination.

The recurrence of inflammation during the tapering treatment process is often a problem not only in AAU but also in the treatment of uveitis. It is not always easy to assess whether inflammation has resolved using slit-lamp microscopy, fundus examination, or OCT. We have reported that retinal functional changes precede the retinal structural changes [11, 14, 16]. We believe that the functional changes of the retina could be used to predict retinal structural changes, and by making treatment decisions based on the extent of these functional changes, we could provide more accurate and customized treatment.

Despite these promising results, this study has several limitations. First, the sample size was small, the follow-up period was relatively short, and the ERG/OCT examinations were performed at irregular time points, limiting evaluation of the association between ERG amplitude changes and CMT. In addition, symptom onset was based on patient self-report, which may have introduced imprecision in the estimated time course and peak timing. Second, because pre-disease baseline data were unavailable, outcomes were normalized to the fellow eye, which assumes comparable baseline values between eyes. Objective inflammatory biomarkers (e.g., laser flare photometry) were not available, limiting our ability to characterize inflammatory activity quantitatively over time. In addition, the treatment intensity (e.g., initial steroid dosing and tapering speed) was individualized based on clinical response and may have influenced the estimated peak timing and recovery trajectories of ERG and CMT. Finally, severe cases of AAU that progressed to clinically apparent CME were not included, limiting inference regarding such cases.

In conclusion, we identified an increase in the flicker ERG amplitudes in eyes with AAU. We also found that the CMT increases slightly later than the increase in ERG amplitude. The use of ERG recordings in eyes with AAU may be valuable for evaluating posterior segment inflammation, which may not be clearly detected through anterior segment examinations or OCT findings. Our findings suggest that flicker ERGs could serve as a useful tool for assessing subclinical inflammation in the posterior segment of eyes with AAU, and its use is expected to enhance the quality of treatment by providing more precise monitoring of retinal inflammation.
